# Assessment of a viral load result-triggered automated differentiated service delivery model for people taking ART in Lesotho (the VITAL study): Study protocol of a cluster-randomized trial

**DOI:** 10.1371/journal.pone.0268100

**Published:** 2022-05-05

**Authors:** Nadine Tschumi, Malebanye Lerotholi, Mathebe Kopo, Mpho Kao, Blaise Lukau, Bienvenu Nsakala, Ntoiseng Chejane, Lipontso Motaboli, Tristan Lee, Ruanne Barnabas, Adrienne E. Shapiro, Alastair van Heerden, Thabo I. Lejone, Alain Amstutz, Jennifer A. Brown, Jesse Heitner, Jennifer M. Belus, Frédérique Chammartin, Niklaus D. Labhardt

**Affiliations:** 1 Swiss Tropical and Public Health Institute, Allschwil, Switzerland; 2 University of Basel, Basel, Switzerland; 3 Ministry of health, Maseru, Lesotho; 4 Solidarmed Lesotho, Maseru, Lesotho; 5 Division of Infectious Diseases, Massachusetts General Hospital, Boston, MA, United States of America; 6 University of Washington, Seattle, Washington, United States of America; 7 Center for Community Based Research, Human Sciences Research Council, Pietermaritzburg, South Africa; 8 Faculty of Health Sciences, SAMRC/WITS Developmental Pathways for Health Research Unit, Department of Paediatrics, School of Clinical Medicine, University of the Witwatersrand, Johannesburg, Gauteng, South Africa; 9 Department of Clinical Research, Basel Institute for Clinical Epidemiology and Biostatistics, University Hospital Basel, University of Basel, Basel, Switzerland; 10 University Hospital Basel, Basel, Switzerland; PLOS: Public Library of Science, UNITED KINGDOM

## Abstract

**Introduction:**

To sustainably provide good quality care to increasing numbers of people living with HIV (PLHIV) receiving antiretroviral therapy (ART) in resource-limited settings, care delivery must shift from a “one-size-fits-all” approach to differentiated service delivery models. Such models should reallocate resources from PLHIV who are doing well to groups of PLHIV who may need more attention, such as those with treatment failure. The VIral load Triggered ART care Lesotho (VITAL) trial assesses a viral load (VL)-, participant’s preference-informed, electronic health (eHealth)-supported, automated differentiated service delivery model (VITAL model). With VITAL, we aim to assess if the VITAL model is at least non-inferior to the standard of care in the proportion of participants engaged in care with viral suppression at 24 months follow-up and if it is cost-saving.

**Methods:**

The VITAL trial is a pragmatic, multicenter, cluster-randomized, non-blinded, non-inferiority trial with 1:1 allocation conducted at 18 nurse-led, rural health facilities in two districts of northern Lesotho, enrolling adult PLHIV taking ART. In intervention clinics, providers are trained to implement the VITAL model and are guided by a clinical decision support tool, the VITALapp. VITAL differentiates care according to VL results, clinical characteristics, sub-population and participants’ and health care providers’ preferences.

**Expected outcomes:**

Evidence on the effect of differentiated service delivery for PLHIV on treatment outcomes is still limited. This pragmatic cluster-randomized trial will assess if the VITAL model is at least non-inferior to the standard of care and if it is cost saving.

**Trial registration:**

The study has been registered with clinicaltrials.gov (Registration number NCT04527874; August 27, 2020).

## Introduction

Differentiated service delivery (DSD) models are intended to sustainably provide and optimize HIV services for the increasing number of people taking antiretroviral therapy (ART) [1–4]. DSD models transition away from “one-size-fits-all” HIV care and instead endorse client-centeredness while aiming to reduce costs and to improve quality of care for over 25 million people living with HIV (PLHIV) in Sub-Saharan Africa [5]. A core component of DSD is to have participants visit the health clinic less often through multi-month dispensing (MMD) for individuals who are stable in order to use the resources and time saved to attend to participants with unstable disease, problems with medication adherence, or other comorbidities requiring care.

Viral load (VL) monitoring provides a broadly available and objective indicator of treatment response and can thus be used as a basis to differentiate intervals between clinic visits, intensity of adherence support and medical follow-up for individual participants [6–8]. Most programs, however, do not exploit the potential VL monitoring offers to differentiate follow-up [8–15] and often VL results are not met by appropriate clinical action [9,16–20]. Electronic health (eHealth) support to health care providers and participants could help to ensure VL results and their clinical significance are known and followed by timely action [21,22]. Based on VL results, additional clinical information and participant preference, a dedicated eHealth tool could serve as a clinical decision support for health care providers and empower participants by sharing essential information through text messages, while helping to avoid unnecessary health facility visits [23–25].

VIral load Triggered ART care Lesotho (VITAL) is an automated DSD model that is informed by VL result, participant preferences, and clinical parameters. It triages participants into groups requiring different levels of care, guided by a clinical decision support tool for health care providers, called the VITALapp. Further, the VITAL model empowers participants through direct information on laboratory results via text messages, individualized health information, individualized adherence and visit reminders, automated phone calls for symptomatic tuberculosis (TB) screening, and the option for participants to request call-backs by an expert nurse in case of concerns or questions.

We present here the protocol of the VITAL trial, a pragmatic 1:1 cluster-randomized trial at 18 ART clinics in Lesotho to assess non-inferiority of the VITAL model to standard of care in terms of engagement with viral suppression.

## Methods

### Setting

Lesotho, a small country surrounded by South Africa, has an estimated adult HIV prevalence of 21%. Over 96% and about 73% of HIV-infected women and men, respectively, were taking ART in 2021 [26]. The trial is conducted at nurse-led clinics in the districts of Butha-Buthe and Mokhotlong in Northern Lesotho that serve a mostly rural population. In 2020, the number of participants actively taking ART per clinic ranged from about 400 to about 1200 participants.

### Design

VITAL is a pragmatic, multicenter, 1:1 cluster-randomized, non-blinded, non-inferiority trial. Each clinic forms a cluster. A cluster design was chosen because of the high risk of cross-contamination between study arms if randomization were done at participant level.

### Eligibility and study participants

Eligible clusters are public or missionary nurse-led health facilities (not hospitals) that offer ART services, serve a mostly rural population, and are situated in an area with stable cell phone signal. People are eligible for inclusion if they are living with HIV, taking ART and registered for HIV care at one of the eligible clusters, aged 18 years and above, intending to remain at the facility for the duration of the trial, and able to provide a formal informed consent. Access to a cell phone is not an eligibility criterion.

### Randomization and blinding

Cluster randomization took place at a meeting with representatives of all health center clusters in each district to increase ownership of the participating health facilities. The representatives sequentially drew opaque (black), equally-sized and sealed envelopes containing the group allocation from a Mokorotlo (traditional Lesotho hat) according to a randomized sequence. Allocation disclosure and documentation thereof only happened after all representatives had drawn their envelope. Randomization was stratified by district due to differences in viral suppression rates between the districts (Butha-Buthe versus Mokhotlong). Participating clinics were assigned (1:1) to either offer standard care or the VITAL model. Although this is an open-label trial, laboratory staff who assess the primary endpoint are blinded.

### Enrolment procedures

**Fig 1:**
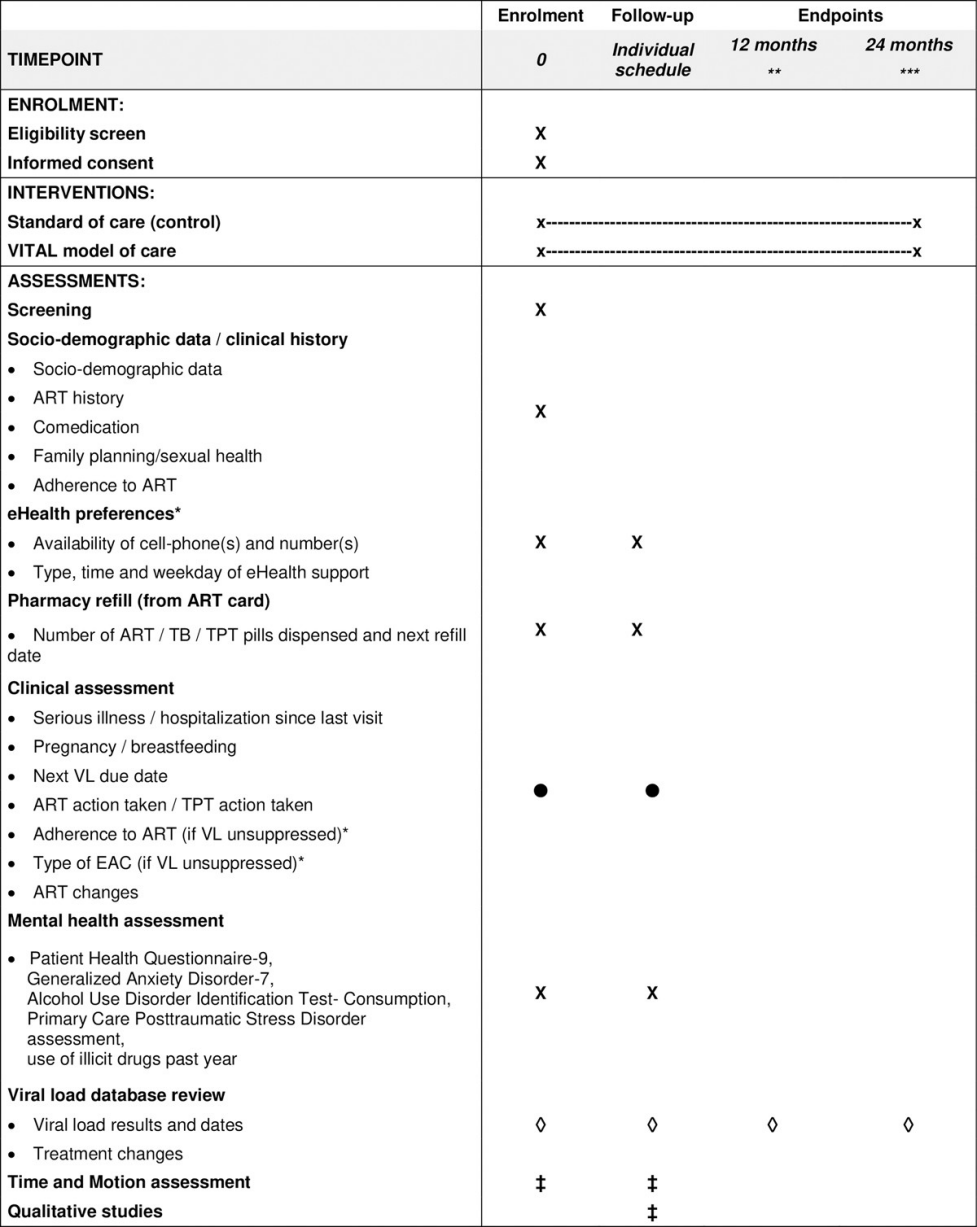
Spirit flow diagram: Schedule of enrolment, interventions, and assessment procedures. Symbols correspond to the responsible person for the assessment or task: X: VITAL study team, •: Clinic staff, ◊: VITAL data management team, ‡: Other VITAL study team members. * in VITAL intervention clinics only; ** 12 months window; *** 24 months window. TB: Tuberculosis; TPT: Tuberculosis preventive therapy; VL: Viral load; EAC: Enhanced adherence counselling.

In both control and intervention clusters, dedicated study staff at each participating clinic actively screen all people seeking health services at the clinic. If eligible after screening, potential participants are approached for informed consent. Illiterate participants provide a thumbprint and choose a literate witness to co-sign the arm-specific consent form. Study staff subsequently interviews participants following a set of questionnaires, including socio-demographic, clinical and treatment adherence information, eHealth preferences (in the intervention clusters only) and mental health functioning. A summary of the schedule of enrolment, interventions and assessment procedures is outlined in Fig 1, following the Standard Protocol Items: Recommendations for Interventional Trials (SPIRIT) recommendations [27]. Participating clinics enroll participants until the sample size per cluster is reached or until decision to stop enrollment was reached.

### Intervention and control

**Fig 2:**
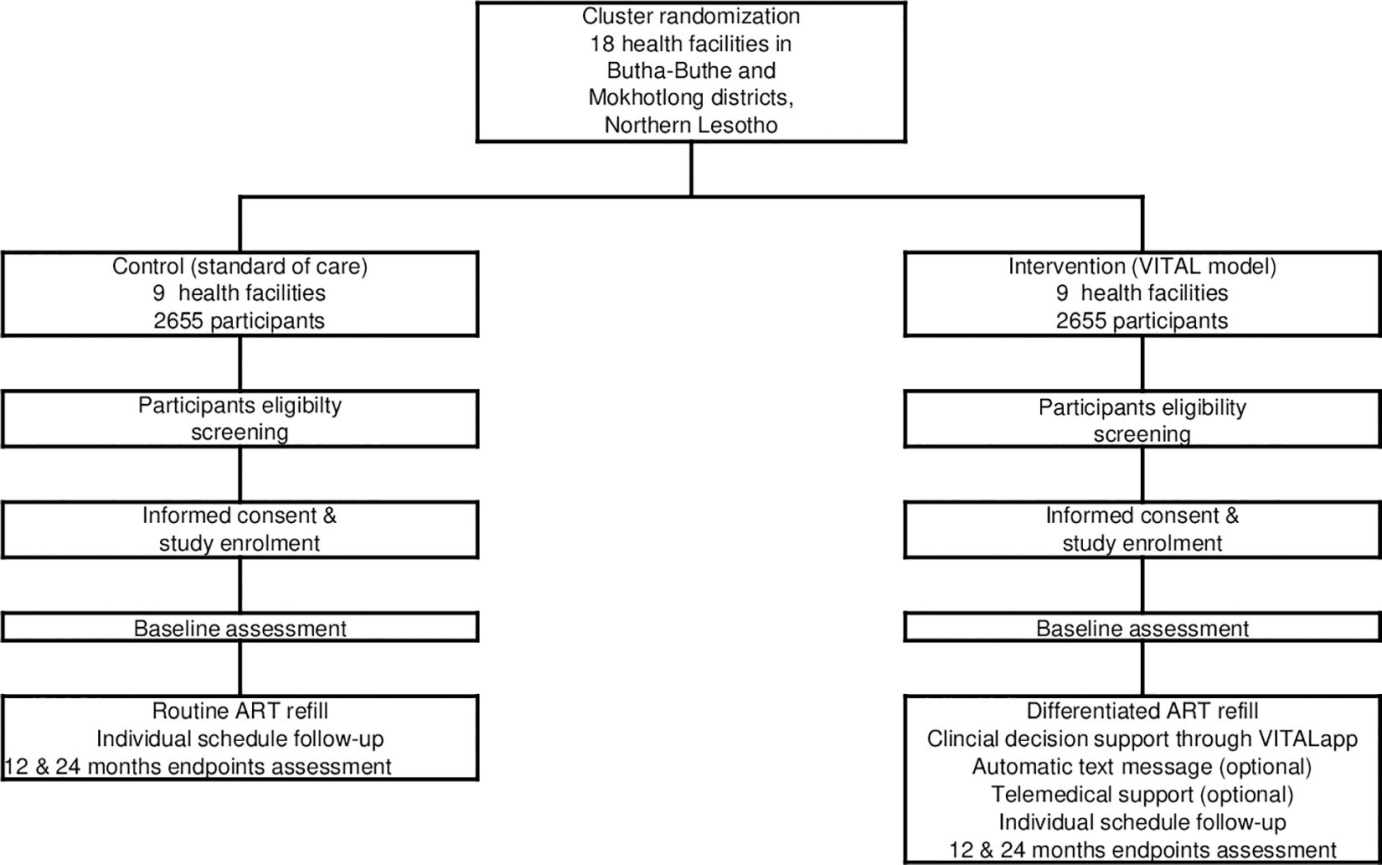
Flow chart of study protocol.

**Fig 3:**
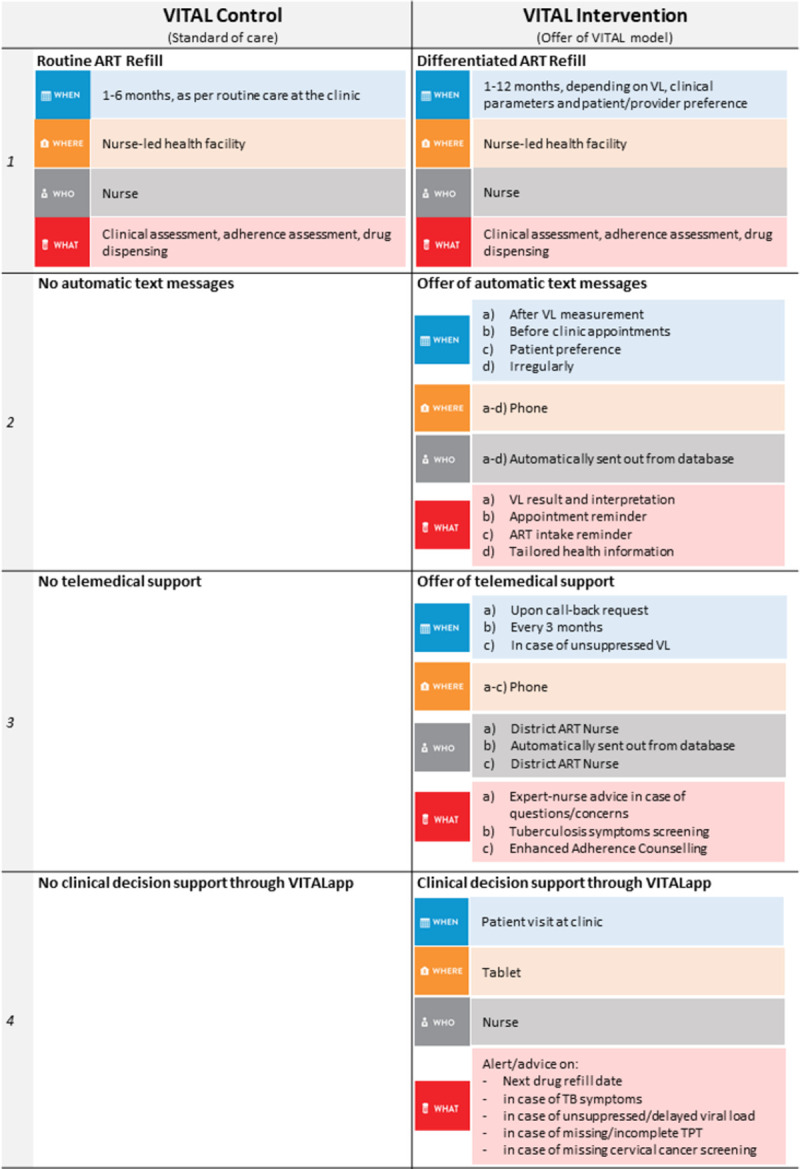
Differentiated service delivery building blocks of the VITAL model. VL: Viral load; TB: Tuberculosis; TPT: Tuberculosis preventive therapy; ART: Antiretroviral therapy.

Participants in the intervention clusters receive services as per the VITAL model. Components of VITAL were developed during a formative pilot study at two health centers in 2019 [28]. VITAL interfaces HIV care at three levels: determination of the interval until the next clinic visit, eHealth support options for participants; and eHealth clinical decision support for the nurses. A flow chart of the study is provided in Figs 2 and 3 shows the DSD building blocks of the VITAL model.

#### Visit interval

To determine the interval to the next clinic visit or drug refill, the VITALapp suggests to the health care provider the longest possible interval (max 12 months), taking into consideration the last VL result, date when the next VL measurement is due, participant preference, and clinical information on comorbidities (TB). The provider may overrule the VITALapp suggestion based on his or her clinical judgement.

#### eHealth support for participants

As eHealth support for participants, the VITALapp offers direct VL result communication via SMS, clinic appointment and ART intake reminders, and tailored health messages with other relevant health-specific information such as reminder of cervical cancer screening. Participants are free to opt in for any of these message services. To ensure confidentiality, participants can choose wording, frequency, and timing of the text messages. All SMS options are worded in a way that does not disclose the recipient’s HIV status. In addition to automated text messages, participants with refill intervals longer than three months receive an automated call screening for clinical signs of TB. In this call, the participant is asked about the four standard TB symptoms (cough, weight loss, night sweats, loss of appetite) and asked to respond by pressing 1 for “yes” and 2 for “no”. In case of positive screening, the participant is advised to attend the clinic for a TB evaluation. Participants with unsuppressed VL at last measurement may opt for adherence counselling and support by an expert nurse via phone instead of having to attend the clinic. Further, all participants may at any time request a call-back from the expert nurse in case they have any questions or medical concerns.

#### eHealth clinical decision support for nurses

For health care providers, the VITALapp creates a detailed overview of the clinical history of the participant, including previous VL results, previous clinical TB screening and TB test results, status with regard to tuberculosis preventive therapy (TPT), and previous cervical cancer screenings. Based on the available laboratory and clinical information, the VITALapp suggests the appropriate actions according to national HIV, TB and cervical cancer screening guidelines. The nurses are, however, able to overrule these recommendations based on their clinical judgement. Further, the VITALapp generates an overview for each clinic, listing all participants with unsuppressed VL, treatment failure, or who are overdue for their clinic appointment.

Participants in the control clusters are offered the standard of care (Fig 3). Before the Coronavirus Disease 2019 (COVID-19) pandemic, ART and TB drug supply (treatment or prevention) was limited to three months. During the pandemic, standard of care started to allow drug refill intervals of up to 6 months. In control clinics, participants’ preferences for clinic visit intervals are not systematically assessed and they do not receive any of the above-mentioned eHealth support options. Nurses in control clinics only have access to a restricted version of the VITALapp that allows for data entry but does not display participant information or treatment recommendations.

At intervention and control clinics, health care services are exclusively provided by clinic-employed nurses. VITAL study staff at these clinics do conduct enrolment of participants and documentation of their follow-up but do not directly interfere with normal procedure health care provision or management of patients at any level.

### Outcomes

The primary endpoint is engagement in care with viral suppression (<20 copies/mL) at 24 months follow-up (16–28 months window). Our pragmatic trial aims to display real-life effectiveness of the VITAL model. Therefore, participants will not be contacted or traced to ensure VL measurement and only standard routine tracing procedures that are already in place will be applied. Participants that do not attend a clinic visit for VL testing, for whom no VL sample was taken, or had no VL measurement for technical reasons, are considered as not having a documented VL within the primary endpoint window. Secondary endpoints are 1) the proportion of deaths at 12 and 24 months after enrollment, 2) the proportion of participants with confirmed TB diagnosis at 12 and 24 months after enrollment, and 3) the proportion of disengagement from care at 12 and 24 months after enrollment, 4) time to follow-up VL in case of an unsuppressed VL (≥ 20 copies/mL), 5) time to switch to a new ART regimen in case of virologic failure, 5) rate of health center visits at 24 months after enrollment, 6) the proportion of participants with ART modification due to virologic failure at 12 and 24 months among participants with virologic failure and 7) the proportion of participants receiving a course of TB preventive therapy. A summary of primary and secondary endpoints, together with their respective research hypotheses, is given in S1 Table.

In addition, we will also measure intervention-cluster specific outcomes such as 1) the proportion of participants requesting a VL result notification through text message, 2) the proportion of text messages delivered successfully, 3) the proportion of participants using the call-back option through the District ART Nurse, 4) the proportion of participants screened positive for TB by automated call, 5) the proportion of participants appreciating the automated differentiated service delivery model and 6) the proportion of health care providers appreciating the automated differentiated service delivery model.

### Assessment of cost

We will analyse cost-effectiveness if the intervention is found to be superior or assess the budget impact if the intervention is found to be non-inferior. For the cost-effectiveness analysis, we will use the primary endpoint and mathematical models of HIV transmission. Costing data will include: (1) conversion rate of local currency to U.S. dollars at 6-month intervals over the life of the project; (2) costs of all commodities used in the intervention; (3) community sensitization, promotional and advertising costs; (4) average time clients spent with intervention including transportation, (5) staff time and representative salaries; (6) local average wages of the target population; (7) remunerations to clinics; and (8) other relevant costs, including training of providers and transport costs.

Time and motion studies are conducted to collect the costing data necessary to provide the intervention. They are conducted over a 2 to 4 week period at a minimum of two random sites during enrolment and again when the intervention is running at full capacity. An experienced research assistant collects data on time required to complete each step of the intervention. Observing multiple visits allows estimation of the average time taken for each step. The time taken for research purposes (e.g. data collection) is noted separately from the estimated time needed for clinical services. In addition, interviews with study staff to quantify the effort required for each step of the intervention are conducted, as well as interviews with participants to assess opportunity costs. We also ask participants what expenses and opportunity costs they incurred while receiving the intervention. Furthermore, we collect data on the average cost of medical care in Lesotho associated with HIV infection and AIDS through literature review. A discount rate of 3% is used with sensitivity analysis of 0% to 5%.

### Data collection and management

Each participating clinic receives two tablets with the VITALapp installed. At each clinic, one tablet is used by dedicated non-clinical study staff to enrol participants and document follow-up. The other tablet is used by the nurse who is seeing participants for HIV care. Clinical and laboratory data from participants are stored in a dedicated database established in both districts since 2015 and described in detail previously [16]. Every time the VITALapp synchronizes with the database, laboratory data from the database and clinical information from the tablets are synchronized. The VITALapp is password protected and the VL database requires two factor authentication. The data are stored in banking-standard ISO 27001-audited data centres in Switzerland and participant names are encrypted. Nurses and study staff at health centres identify participants only by entering their unique national ART number. Data are monitored centrally and checked regularly by the data-management team of the Swiss Tropical and Public Health Institute. Weekly reports are issued to the study teams, highlighting potential data-inconsistencies that are then followed-up by the VITAL study data-managers in Lesotho.

### Trial monitoring and severe adverse events

The trial will be monitored by the Clinical Operations Unit of the Swiss Tropical and Public Health Institute. Because VITAL trial uses treatments and drug-doses as per international and national guidelines, all treatment components will be applied at standard dosage and no new substances or alternative indications will be tested, a risk-based monitoring approach will be applied, focusing on potential Severe Adverse Events (SAE). Health care providers in charge at a study facility report SAE to the local principal investigator who informs the principal investigator and the sponsor/chief investigator. In case a causal relationship of the SAE with the VITAL trial is judged as possible, the National Health and Research Ethics Committee (NH-REC) of Lesotho is informed within 72 hours. SAEs judged as not or unlikely to be related to the VITAL trial are attached to the annual interim report submitted to NH-REC. Site monitoring will be conducted by trained monitors of SolidarMed, under supervision of the Clinical Operations Unit. In addition, monitors from Clinical Operations Unit will conduct site visits at least once per year. The trial does not have a data monitoring committee but 6-monthly meetings of the steering committee where recruitment status and occurrence of SAEs are reported and discussed. Further, study status and SAEs are reported on a yearly basis to the NH-REC. No interim analysis is planned.

### Sample size and statistical analysis

#### Non-inferiority margin

We chose a margin of non-inferiority for the odds ratio (OR) of reaching our primary endpoint of engagement in care with documented viral suppression of 0.8. On an absolute scale, this non-inferiority margin corresponds to a higher absolute probability of failing to reach the primary endpoint of 5% in the intervention compared to the control group, assuming that 65% per cent of participants in the control arm will be engaged in care with documented viral suppression at the end of the follow-up period [16].

#### Sample size

Sample size is calculated assuming an individual randomization inflated by a design effect that account for variation at cluster level [29–32], according to the code developed by Rotondi and Donner [30]. Assuming an intra-cluster correlation of 0.04, a mean cluster size of 347 participants (standard deviation = 128), a probability of success in the intervention group of 75% and a probability of success in the control group of 65%, a sample size of 5310 participants (2655 participants in each arm) is required for a type I error of 0.025 and a statistical power of 80%. We thus decide to enrol 18 clinics, 9 per arm, aiming for a total of 6246 participants which exceed the calculated sample size of 5310.

#### Statistical analysis

Participants will be analysed according to their cluster allocation arm (intention-to-treat (ITT)). Baseline participants’ characteristics and intervention-cluster specific outcomes will be summarized as frequency and percentage with 95% confidence interval, or median with interquartile ranges. VL suppression at 24 months [8–15,23,33,34] after enrolment (primary outcome) will be modelled using a mixed-effect logistic regression model, where cluster variation is accounted as a random effect. Non-inferiority of the intervention arm will be concluded if the lower bound of the 95% CI of the OR of intervention is superior to 0.8 (non-inferiority margin). Non-inferiority and superiority will be concluded if the OR is superior to one and the 95% CI does not include 1. Our model will be further adjusted for socio-demographics characteristics (age, gender, education and employment status, HIV testing history) that are suspected to be associated to the primary endpoint in order to assess their effect on the primary endpoint. Participants with missing clinical outcomes will be dropped from the respective clinical assessments, with the exception of the primary endpoint for which missing information are considered as a failure to be engaged in care. Participants’ characteristics of the dropped population will be compared to patient characteristics of the trial participants that have an outcome to avoid further bias. Missing baseline participants’ characteristics will be imputed using multiple imputation techniques in subsequent analyses.

Clinically very important secondary endpoints are listed separately, will be evaluated independently of the primary objective and would require further investigation if significant differences were observed, but the primary objective was not been achieved. As for the primary endpoint analysis, estimated OR from a mixed-effect logistic regression for intervention will be reported. Continuous secondary endpoints will be reported as mean with 95% Wald CI for each treatment arm, as well as difference in means between participants allocated to the standard or care and participants allocated to the intervention arm.

A detailed statistical analysis plan will be developed before the last participant completes follow-up and will be added to the study protocol at clinicaltrials.gov. Deviations from the statistical plan will be described and justified in the final trial report.

#### Subgroup and sensitivity analysis

We will restrict our trial population to participants with unsuppressed VL (≥ 20 copies/mL) during the first 12 months of follow-up and analyse 1) the proportion of participants with viral re-suppression (<20 copies/mL) 24 months (16–28 months) after enrolment and 2) the proportion with sustained VL suppression (defined as >1 VL <20 copies/mL) during 24 months (16–28 months) follow-up. Robustness to the VL threshold at 20 copies/mL will be addressed in sensitivity analyses where the threshold will be increased to 1000 copies/mL. The rationale for modifying the VL threshold is that some clinics might shift to point-of-care VL measurement or VL measurement from dry blood spot and these approaches have a higher threshold of detection.

### Communication of important protocol amendments

Substantial changes to the study setup and study organisation, the protocol and relevant study documents will be submitted to the Ethics Committee in Lesotho for approval before implementation. Under emergency circumstances, deviations from the protocol to protect the rights, safety and well-being of human subjects may proceed without prior approval of the Ethics Committee. Such deviations shall be documented and reported to the Ethics Committee in Lesotho as soon as possible. A list of all non-substantial amendments will be submitted once a year as part of the annual study progress report to the Ethics Committee in Lesotho.

### Dissemination plans

Results will be reported according to the CONSORT guidelines for cluster-randomized trials [35] and study findings will be submitted for peer-review in open-access journal. Study authorship will follow International Committee of Medical Journal Editors recommendations and we do not intend to use professional writers.

### Trials within cohort and nested studies

The VITAL trial will allow for building a well-documented cohort of adult participants taking ART at 18 clinics. This cohort will provide a platform for multiple future nested cohort trials. Such trials will be developed within specific additional protocols and will be registered separately.

We further foresee several observational nested studies. In a first study, we will describe uptake and completion of TB preventive therapy, as well as diagnosis and treatment of active TB. In a second sub-study, a subsample of VITAL participants in both arms will undergo mental health quantitative and qualitative assessment at enrolment and one-year follow-up, given the documented high burden of mental health problems in PLHIV [36] and its effect on HIV care [37–40]. Quantitative assessments will include the Patient Health Questionnaire-9, the Generalized Anxiety Disorder-7, Primary Care Posttraumatic Stress Disorder assessment, and the Alcohol Use Disorder Identification Test- Consumption to assess depressive symptoms, anxiety and trauma symptoms, and alcohol consumption, respectively. In a third sub-study, we aim to assess virologic outcomes and emergence of resistance after introduction of dolutegravir-based ART. Finally, we aim to describe uptake of cervical cancer screening among female participants.

## Discussion

The VITAL trial is a pragmatic open-label cluster randomized trial in Lesotho to assess the VITAL model in terms of engagement in care and viral suppression among adult PLHIV taking ART. Several studies, and experience during the COVID-19 pandemic, have shown that for participants with viral suppression, longer intervals between clinic visits are non-inferior to standard of care [17,33,34,41]. The VITAL model of care, however, not only focuses on MMD for participants with viral suppression but involves all participants taking ART, while considering participant preference, laboratory and clinical parameters. Through various eHealth options including direct communication of VL results, it aims to empower participants, with text messages and calls to maintain a connection between participants and the health care system, allowing for longer intervals between clinic visits. The VITAL model of care is coordinated through the VITALapp, which assists the health care providers as a clinical decision support tool. Apart from the expert nurse available for call-back, the VITAL model does not foresee any additional human resources, making it a potentially scalable DSD model. We have chosen a pragmatic design where all care is provided by routine clinical staff and clinical and laboratory monitoring follows the established routine at the study clinics.

The VITAL trial has several limitations. First, due to its pragmatic design, the protocol does not define a schedule for study-specific VL testing but rather allows participants to follow their individual routine VL monitoring schedule. As a result, the endpoint windows are wide. Second, for participants newly initiated on ART or transferred in from another district (and therefore without previous VL data in our database), the VITALapp will not be able to disclose the laboratory information to inform providers and participants of actionable care steps. Third, due to the nature of the intervention, participant and provider blinding is not feasible. Fourth, regardless of arm, there may be a considerable Hawthorne effect with health care providers at participating facilities being motivated to perform particularly well knowing that data on their consultations are collected. Finally, the VITAL trial started during the COVID-19 pandemic, which introduced MMD up to six-months as standard of care. This is likely to decrease the difference in visit frequency between control and intervention arm.

Despite these limitations, the VITAL trial will be among the first larger-scale trials to assess a potentially scalable DSD model that not only promotes MMD, but also assesses the clinical effectiveness of eHealth supported DSD for participants with and without suppressed VL. The model takes into account participant preference, clinical conditions, and laboratory results in a pragmatic close to real-life design.

## Supporting information

S1 Table Overview of primary and secondary endpoints and hypotheses. (DOCX)

S1 File SPIRT check list. (DOC)

S2 File Inclusivity in global research. (DOCX)

## Abbreviations

DSD: differentiated service delivery
ART: antiretroviral therapy
PLHIV: people living with HIV
MMD: multi-month dispensing
VL: viral load
eHealth: electronic health
VITAL: Viral load triggered antiretroviral therapy care Lesotho
TB: tuberculosis
SPIRIT: Standard Protocol Items: Recommendations for Interventional Trials
TPT: tuberculosis preventive therapy
COVID-19: coronavirus disease 2019
SAE: severe adverse events
NH-REC: national health and research ethics committee
OR: odds ratio
ITT: intention-to-treat
