# Supplementary material for: Assessment of a viral load result-triggered automated differentiated service delivery model for people taking ART in Lesotho (the VITAL study): Study protocol of a cluster-randomized trial
Source: PLoS One. 2022 May 5;17(5):e0268100. doi: 10.1371/journal.pone.0268100 (PMC9071137; doi:10.1371/journal.pone.0268100)
Supplement: S1 Table — (DOCX) [file pone.0268100.s001.docx]

Table 1. Overview of primary and secondary endpoints and hypotheses.

| **Primary endpoint** | **Research hypothesis** |
| --- | --- |
| 1. Proportion of participants engaged in care (defined as documented visit attendance) with documented viral suppression (<20 copies/mL) 24 months (16-28 months) after enrollment | Intervention arm is non-inferior (non-inferior and superior) |
| **Secondary endpoints indicative of clinical benefit or harm** |  |
| 1. Proportion of death at 12 and 24 months after enrollment 2. Proportion of death at 12 and 24 months after enrollment 3. Proportion of participants with confirmed TB diagnosis at 12 and 24 months after enrollment 4. Proportion of disengagement from care at 12 and 24 months after enrollment   **Secondary endpoints expressing supportive evidence**   1. Time to follow-up VL in case of an unsuppressed VL (≥ 20 copies/mL) 2. Time to switch to a new ART regimen in case of virologic failure 3. Rate of health center visits at 24 months after enrollment 4. Proportion of participants with ART modification due to virologic failure at 12 and 24 months among participants with virologic failure 5. Proportion of participants receiving a course of TB preventive therapy | No difference  No difference  Lower in intervention clusters  Shorter in intervention clusters  Shorter in intervention clusters  Higher in intervention clusters  No difference  Higher in intervention clusters |
| Months are defined as calendar months. If multiple measurement are available within a specific time window, the closest measurement for the target point will be used for analysis.  ART: antiretroviral therapy; VL: viral load; TB: tuberculosis | |
